# A Survey of Topological Machine Learning Methods

**DOI:** 10.3389/frai.2021.681108

**Published:** 2021-05-26

**Authors:** Felix Hensel, Michael Moor, Bastian Rieck

**Affiliations:** ^1^Machine Learning and Computational Biology Laboratory, ETH Zurich, Zurich, Switzerland; ^2^Swiss Institute of Bioinformatics, Lausanne, Switzerland

**Keywords:** computational topology, persistent homology, machine learning, topology, survey, topological machine learning

## Abstract

The last decade saw an enormous boost in the field of computational topology: methods and concepts from algebraic and differential topology, formerly confined to the realm of pure mathematics, have demonstrated their utility in numerous areas such as computational biology personalised medicine, and time-dependent data analysis, to name a few. The newly-emerging domain comprising topology-based techniques is often referred to as topological data analysis (TDA). Next to their applications in the aforementioned areas, TDA methods have also proven to be effective in supporting, enhancing, and augmenting both classical machine learning and deep learning models. In this paper, we review the state of the art of a nascent field we refer to as “topological machine learning,” i.e., the successful symbiosis of topology-based methods and machine learning algorithms, such as deep neural networks. We identify common threads, current applications, and future challenges.

## 1. Introduction

Topological machine learning recently started to emerge as a field at the interface of topological data analysis (TDA) and machine learning. It is driven by improvements of computational methods, which make the calculation of topological features (via persistent homology, for instance) increasingly flexible and scalable to more complex and larger data sets.

Topology is colloquially often referred to as encoding the overall shape of data. Hence, as a complement to localised and generally more rigid geometric features, topological features are suitable to capture multi-scale, global, and intrinsic properties of data sets. This utility has been recognised with the rise of TDA, and topological information is now generally accepted to be relevant in the context of data analysis. Numerous works aim to leverage such information to gain a fundamentally different perspective on their data sets. We want to focus on a recent “outgrowth” of TDA, i.e., the integration of topological methods to *enhance* or *augment* both classical machine learning methods and deep learning models.

Our survey therefore discusses this ongoing synthesis of topology and machine learning, giving an overview of recent developments in the field. As an emerging research topic, topological machine learning is highly active and rapidly developing. Our survey is therefore explicitly not intended as a formal and complete review of the field. We rather want to identify, present, and discuss some of the main directions of developments, applications, and challenges in topological machine learning as we perceive it based on our own research background. Our aim is to provide newcomers to the field with a high-level overview of some of the central developments and techniques that have been developed, highlighting some “nuggets,” and outlining common threads and future challenges. We focus on publications in major machine learning conferences (such as AISTATS, ICLR, ICML, and NeurIPS) and journals (such as JMLR) but want to note that the selection of topics and papers presented here reflects our own preferences and knowledge. In particular, we decided against the inclusion of unpublished work in this area.

The survey is broadly structured as follows: we first provide a brief mathematical background on persistent homology, one of the core concepts of topological data analysis, in section 2. Following the introduction, the main part of the survey is in section 3. Section 3.2 focuses on what we term *extrinsic topological features* in machine learning. These methods are mainly concerned with the transformation of topological descriptors of data into feature vectors of fixed dimensionality, permitting their use as features in machine learning frameworks. This is in contrast to *intrinsic topological features*, portrayed in section 3.3, which employ topological features to analyse or influence the machine learning model itself, for instance by architectural choices or regularisation. Finally, section 4 discusses future directions and challenges in topological machine learning.

## 2. Background on Algebraic Topology and Persistent Homology

This section provides some background on basic concepts from algebraic topology and persistent homology. For in-depth treatments of the subject matter, we refer to standard literature (Bredon, 1993; Hatcher, 2000; Edelsbrunner and Harer, 2010). Readers familiar with algebraic topology and the concept of persistent homology may safely skip this section.

A basic hypothesis in data analysis which drives current research is that data has *shape*, or put differently, that data is sampled from an underlying manifold—the so-called “manifold hypothesis” (Fefferman et al., 2013). Instead of restricting the analysis to statistical descriptors, *topological data analysis* (TDA) aims to analyse data from a fundamentally different perspective by investigating this underlying manifold structure in an algebraic fashion. Namely, one computes descriptors of data sets which are *stable* under perturbation and encode intrinsic *multi-scale* information on the their shape. TDA is a rapidly developing field of mathematics aiming to leverage concepts of the well-established field of (algebraic) topology toward applications for real-world data sets and machine learning.

Topology studies invariant properties of (topological) spaces under homeomorphisms (i.e., continuous transformations); in the following, we restrict ourselves to topological manifolds, so as to simplify the exposition. A fundamental problem in topology is about classification: *How can two manifolds be distinguished from each other?* Algebraic topology (Bredon, 1993; Hatcher, 2000) provides sophisticated and powerful tools to study this question. The basic idea being to associate computable *algebraic structures* (e.g., groups or vector spaces) to a manifold that remain *invariant* under homeomorphisms. A very important class of algebraic invariants are the *homology groups*, which encode a great deal of information while still being efficiently computable in many cases. Homology groups arise from combinatorial representations of the manifold, the *chain complexes*.

### 2.1. Chain Complexes and Homology

The *standard* *k-simplex* Δ^*k*^ is defined as the convex hull of the standard basis vectors in ℝ^*k*+1^, i.e.,

$$\Delta^{k}\;:\;=\left\{\left(x_{0},\ldots ,x_{k}\right)\in {\mathbb{R}}^{k+1}|\overset{k}{\underset{i=0}{\sum }}x_{i}=1,\;x_{i}\geq 0\;\forall i\right\}.$$

**Figure 1:**
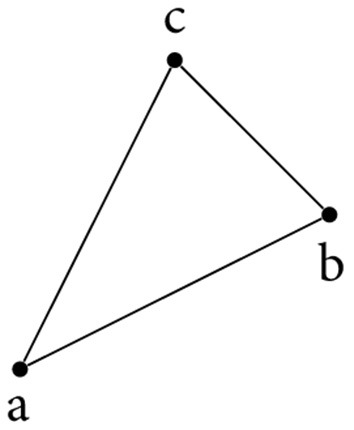
A simplicial complex modelling a triangle.

Similarly, a general *k-simplex* [*v*_0_, …, *v*_*k*_] is the convex hull of *k* + 1 affinely independent points *v*_0_, …, *v*_*k*_ in a Euclidean space. Note that deleting one of the *vertices* *v*_*i*_ from a *k*-simplex [*v*_0_, …, *v*_*k*_] yields a (*k* − 1)-simplex $\left[v_{0},\ldots ,{\hat{v}}_{i},\ldots ,v_{k}\right]$ which is determined by the remaining vertices and called the *i-th face* of [*v*_0_, …, *v*_*k*_]. Simplices are the basic building blocks of chain complexes that are used in algebraic topology for the computation of homological invariants. Any *topological manifold* *X* can be topologically modelled using simplices (see Figure 1). A *singular* *k-simplex* in *X* is a continuous map σ:Δ^*k*^ → *X*. It is not required that σ is an embedding, for instance any constant map, mapping so a single point in *X* is a valid singular simplex. The inclusion of the *i*-th face of Δ^*k*^ is an important singular simplex in Δ^*k*^, which we will denote by $F_{i}^{k}:\Delta^{k-1}\to \Delta^{k}$. To keep the exposition simple we will restrict ourselves to working over the two element field 𝔽_2_: = ℤ/2ℤ in what follows. Given any space *X*, its *singular* *k-chains* are elements of the 𝔽_2_-vector space *C*_*k*_(*X*) generated by the set of all singular *k*-simplices in *X*. Elements in *C*_*k*_(*X*) are thus “formal sums” of simplices. The *singular chain complex* (*C*(*X*), ∂) of *X* is the sequence of spaces

$$\ldots \overset{\partial_{d+1}}{\to }C_{d}\left(X\right)\overset{\partial_{d}}{\to }C_{d-1}\left(X\right)\overset{\partial_{d-1}}{\to }$$

$$\ldots \overset{\partial_{2}}{\to }C_{1}\left(X\right)\overset{\partial_{1}}{\to }C_{0}\left(X\right)\overset{\partial_{0}}{\to }0,$$

together with the *boundary maps* ∂_*k*_ : *C*_*k*_(*X*) → *C*_*k*−1_(*X*) given by

$$\partial_{k}\left(\sigma \right)\;:\;=\underset{i}{\sum }\sigma ◦F_{i}^{k}$$

on the basis elements and extended linearly. A crucial property of the boundary maps is that they compose to 0, that is ∂_*k*_ ◦ ∂_*k*−1_ = 0. Elements of *Z*_*k*_(*X*): = ker(∂_*k*_) are called *k-cycles* and those of *B*_*k*_(*X*): = im(∂_*k*+1_) are called *k-boundaries* and their well-defined quotient

$$H_{k}\left(X\right)\;:\;=Z_{k}\left(X\right)/B_{k}\left(X\right)$$

is the *k-th singular homology group* of *X* (despite the name, this is still technically a quotient vector space; however, the group-theoretical viewpoint is more convenient and prevalent in algebraic topology). The homology groups are *topological invariants*, i.e., they remain invariant under homeomorphisms and therefore encode intrinsic information on the topology of *X*. Thus, homology groups and simpler invariants derived from them, such as the *Betti-numbers* β_*k*_: = dim *H*_*k*_(*X*), are useful in studying the classification question raised above. For example, the 0-th Betti number β_0_ is a count of the connected components of a space, while β_1_ is a count of the number of cycles.

#### 2.1.1. Brief Example

Using the simplicial complex in Figure 1, we briefly illustrate some of the aforementioned concepts. Let *X* = {{*a*}, {*b*}, {*c*}, {*a, b*}, {*b, c*}, {*a, c*}, {*a, b, c*}} be the representation of the simplicial complex. The *boundary* of the triangle is non-trivial, i.e., ∂_2_{*a, b, c*} = {*b, c*} + {*a, c*} + {*a, b*} The boundary of this chain of edges is trivial, though, because duplicate simplices cancel each other out. We get ∂_1_({*b, c*} + {*a, c*} + {*a, b*}) = {*c*} + {*b*} + {*c*} + {*a*} + {*b*} + {*a*} = 0, which is consistent with the property of compatible boundary maps to compose to 0. To compute *H*_1_(*X*): = *Z*_1_(*X*)/*B*_1_(*X*), we only have to calculate *Z*_1_(*X*); the boundary group *B*_1_(*X*) does not contain any non-trivial simplices because *X* does not contain any 2-simplices. By definition, *Z*_1_(*X*) = ker(∂_1_) = span ({*a, b*} + {*b, c*} + {*a, c*}). This is the *only* cycle in *X* (which we can easily verify either by inspection or based on combinatorics). Hence *H*_1_(*X*) = *Z*_1_(*X*) = 𝔽_2_ and β_1_ = 1; the triangle therefore exhibits a single cycle, which aligns with our intuition.

### 2.2. Persistent Homology

Persistent homology (Edelsbrunner et al., 2000; Zomorodian and Carlsson, 2005) is the flagship tool of TDA. In the analysis of real-world data, it is typically not a priori clear at what *scale* interesting topological features occur. By using a filtration (connected to the scale parameter) persistent homology is able to capture topological changes across the whole range of scales and store this information in so-called persistence diagrams.

**Figure 2:**
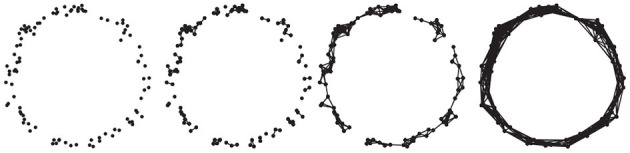
Different stages of a Vietoris–Rips filtration for a simple “circle” point cloud. From left to right, connectivity of the underlying simplicial complex increases as ϵ increases.

*Persistent homology* is an extension of homology to the setting of filtered chain complexes. A *filtered chain complex* is a (not-necessarily strictly) ascending sequence of chain complexes $C^{\varepsilon_{0}}\subset C^{\varepsilon_{1}}\subset C^{\varepsilon_{2}}\subset \ldots$ with inclusion maps *ι*^*i*^ : *C*^*ε*_*i*_^
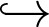
*C*^*ε*_*i*+1_^ and $\iota^{i,j}:=\iota^{j}◦\iota^{j-1}◦\cdots ◦$ *ι*^*i*^ : *C*^*ε*_*i*_^
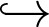
*C*^*ε*_*j*_^ for *i* < *j*. Filtered chain complexes naturally arise in situations where we have a sequence of inclusions of spaces $X^{\varepsilon_{0}}\subset X^{\varepsilon_{1}}\subset X^{\varepsilon_{2}}\subset \ldots$. Such cases, for instance, occur if we consider the sublevel sets $X^{\varepsilon }:=f^{-1}\left({\mathbb{R}}_{<\varepsilon }\right)$ of a so-called *filtration* function *f* : *X* → ℝ, or if we consider a point cloud *Y* in a metric space (*M*, d) and set

$$Y^{\varepsilon }\;:\;=\underset{y\in Y}{⋃}B_{\varepsilon }\left(y\right)=g^{-1}\left({\mathbb{R}}_{<\varepsilon }\right)$$

with filtration function *g* : *M* → ℝ given by $g\left(m\right):=\inf_{y\in Y}\text{d}\left(m,y\right)$. Here *B*_ε_(*y*) denotes the open ball of radius ε centred at *y* and we implicitly identify ε ≃ ε′ if *X*^ε^ (resp. *Y*^ε^) is canonically homeomorphic to *X*^δ^ (resp. *Y*^δ^) for all δ ∈ [ε, ε′]. An important property of (singular) homology is that it is *functorial* (see e.g., Bredon, 1993), which implies that the inclusion maps ι^*i, j*^ induce maps on the respective homology groups $H_{k}\left(\iota^{i,j}\right):H_{k}\left(C^{\varepsilon_{i}}\right)\to H_{k}\left(C^{\varepsilon_{j}}\right)$. Figure 2 depicts the Vietoris–Rips complex construction based on a distance filtration, a standard construction in TDA. The *k-th persistent homology groups* are the images of these inclusions, that is

$$H_{k}^{i,j}\;:\;=\text{im }H_{k}\left(\iota^{i,j}\right)=Z_{k}\left(C^{\varepsilon_{i}}\right)/\left(B_{k}\left(C^{\varepsilon_{j}}\right)\cap Z_{k}\left(C^{\varepsilon_{i}}\right)\right),$$

and thus precisely consist of the *k*-th homology classes of $C^{\varepsilon_{i}}$ that still exist after taking the inclusion $H_{k}\left(\iota^{i,j}\right)$. A homology class $\alpha \in H_{k}\left(C^{\varepsilon_{i}}\right)$ is said to be *born* at $C^{\varepsilon_{i}}$ if $\alpha \notin H_{k}^{i-1,i}$, i.e., if it is not in the image of $H_{k}\left(\iota^{i-1,i}\right)$. If α is born at $C^{\varepsilon_{i}}$, it is said to *die* at $C^{\varepsilon_{j}}$ if $H_{k}\left(\iota^{i,j-1}\right)\left(\alpha \right)\notin H_{k}^{i-1,j-1}$ and $H_{k}\left(\iota^{i,j}\right)\left(\alpha \right)\in H_{k}^{i-1,j}$. The *persistence* of α is given by ε_*j*_ − ε_*i*_ and set to infinity if it never dies. The *persistent Betti-numbers*, defined by $\beta_{k}^{i,j}:=\dim H_{k}^{i,j}$, carry information on how the homology (and thus the topology) changes across the filtration.

**Figure 3:**
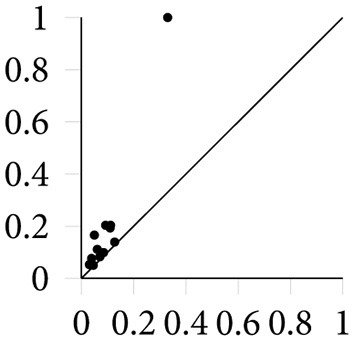
A persistence diagram containing 1-dimensional topological features (cycles).

This information can be captured in a so-called *persistence diagram*, a multiset in ${\bar{\mathbb{R}}}^{2}:={\mathbb{R}}^{2}\cup \mathbb{R}\times \left\{\infty \right\}$. Specifically, the persistence diagram of (homological) *dimension* *k* is given by the points $\left(\varepsilon_{i},\varepsilon_{j}\right)\in {\bar{\mathbb{R}}}^{2}$ with multiplicity

$$\mu_{k}^{i,j}\;:\;=\left(\beta_{k}^{i,j-1}-\beta_{k}^{i,j}\right)-\left(\beta_{k}^{i-1,j-1}-\beta_{k}^{i-1,j}\right)$$

for all *i* < *j*. The multiplicity $\mu_{k}^{i,j}$ counts the number of *k*-th homology classes that are born at $C^{\varepsilon_{i}}$ and die at $C^{\varepsilon_{j}}$. Figure 3 depicts a simple persistence diagram, calculated from the Vietoris–Rips complex in Figure 2. The axes of this diagram correspond to the ϵ values at which topological features are created and destroyed, respectively. The single point of high persistence corresponds to the primary topological feature of the point cloud, namely its circular shape. Other topological features occur at smaller scales—lower values of ϵ—and hence form a small dense cluster in the lower-left corner of the persistence diagram. The persistent Betti-numbers can be recovered from the persistence diagram itself; see Edelsbrunner and Harer, 2010.

A crucial fact that makes persistent homology valuable for application in data analysis is its *stability with respect to perturbations* of the filtration function. This means that persistent homology is robust to noise and constitutes an encoding of intrinsic topological properties of the data. More precisely, the space of persistence diagrams can be endowed with a metric induced by the *bottleneck distance* (or the *Wasserstein distances*) Edelsbrunner and Harer, 2010. A celebrated stability theorem (Cohen-Steiner et al., 2007) states that the *L*_∞_-distance of two real-valued functions *f* and *g* is an upper bound for the bottleneck distance *W*_∞_ of their respective persistence diagrams $D_{f}$ and $D_{g}$, i.e., $W_{\infty }\left(D_{f},D_{g}\right)\leq ||f-g|{|}_{\infty }.$ The stability theorem and its variants (Skraba and Turner, 2020) are highly relevant for applications because they imply that the behaviour of persistent homology under noise is known; descriptors such as persistence diagrams change continuously as the input function is varied, and the “amplitude” of their change is bounded from above via the stability theorem.

## 3. Survey

**Figure 4:**
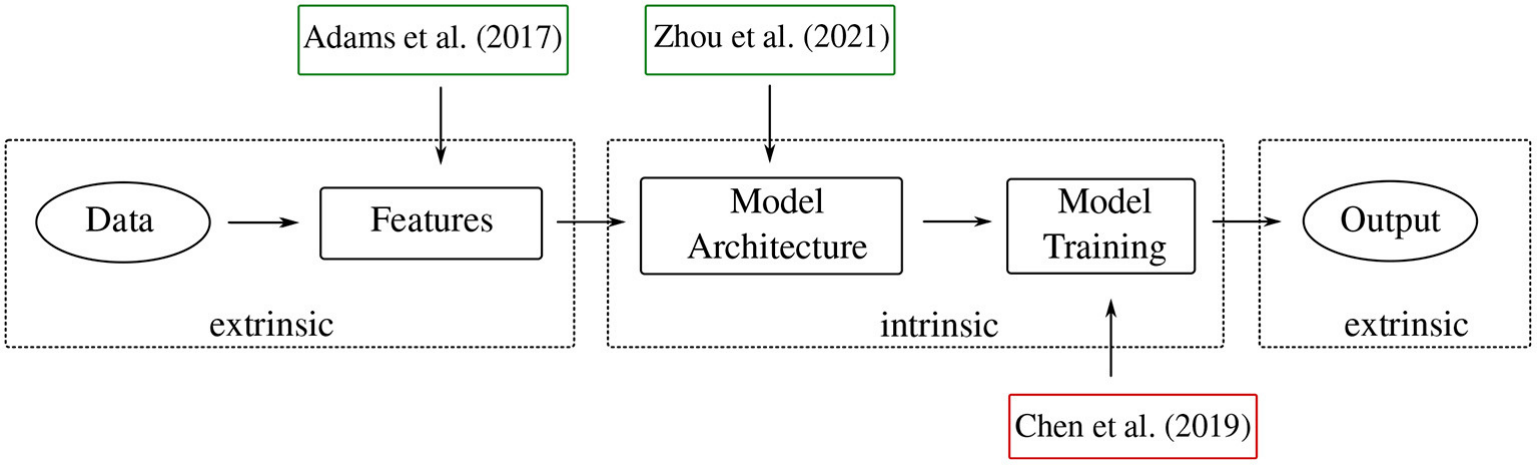
This overview figure shows examples of methods discussed in the survey and their range of influence. Green (red) boxes signify *observational* (*interventional*) methods. Table 1 provides a more in-depth classification of all methods.

This section comprises the main part of the paper, where we gather and discuss pertinent methods and tools in topological machine learning. We broadly group the methods into the following categories. First, in section 3.2, we discuss methods that deal with *extrinsic topological features*. By the qualification *extrinsic*, we mean that no analysis of the topology of the machine learning model or the neural network itself is incorporated. These methods are instead mainly concerned with enabling the use of topological features, extracted from a given data set, in downstream machine learning models. This can be achieved through *vectorisation* of topological features or by designing specialised layers of neural networks that are capable of handling such features. Next, section 3.3 discusses *intrinsic topological features*. Those are methods that incorporate the topological analysis of aspects of the machine learning model itself. Whenever applicable, we further classify methods into *observational* and *interventional* methods. This sub-classification specifies *how* the methods are applied in a machine learning framework. Observational methods “observe” the topology of the data or model but they do not *directly* influence the model training or architecture. Interventional methods, by contrast, apply topological properties of the data, as well as *post-hoc* analysis of topological features of machine learning models, in order to inform the architectural design and/or model training. See Figure 4 for an overview of the methods and their categories, as well as Table 1 for the classification of all papers mentioned in this survey.

**Table 1:** The categorisation of the approaches discussed in the present survey.

**Extrinsic**		**Intrinsic**	
**Observational**	**Interventional**	**Observational**	**Interventional**
Adams et al., 2017	Carrière et al., 2020	Gabrielsson and Carlsson, 2019	Chen et al., 2019
Bubenik, 2015	Kim et al., 2020	Khrulkov and Oseledets, 2018	Hofer et al., 2017
Carrière et al., 2015	Zhao and Wang, 2019	Zhou et al., 2021	Hofer C. et al., 2019
Carrière et al., 2017			Hofer et al., 2020a
Kusano et al., 2018			Hofer et al., 2020b
Reininghaus et al., 2015			Moor et al., 2020
Rieck et al., 2020a			Ramamurthy et al., 2019
Rieck et al., 2020b			Rieck et al., 2019b
Umeda, 2017			Zhao et al., 2020

*It is interesting to note that intrinsic features tend to be used more in interventional settings, whereas extrinsic features remain observational for the most part*.

### 3.1. Limitations

Our paper selection is a cross-section over major machine learning conferences and machine learning journals. We refrain from comparing methods on certain tasks—such as classification—because there is considerable heterogeneity in the experimental setup, precluding a *fair* assessment of such methods.

### 3.2. Extrinsic Topological Features in Machine Learning

This section gives an overview of methods that aim at suitably representing topological features in order to use them as input features for machine learning models. We will refer to this class of methods as *extrinsic topological features in machine learning*, as they take topological information of the data sets into account, as opposed to intrinsic topological information of the machine learning framework itself (see section 3.3). A large class of such methods is comprised of *vectorisation* methods, that aim to transform persistent homology information into a feature vector form in order to make use of it in machine learning models. However, alternative representations of topological descriptors, such as kernels or function-based representations, are also discussed in this section.

#### 3.2.1. Vector-Based and Function-Based Representations

Persistence diagrams (see section 2) constitute useful descriptors of homological information of data. However, being multisets, they cannot be used *directly* as input data for machine learning models in the usual sense (recent paradigm shifts in machine learning, namely the introduction of *deep sets* (Zaheer et al., 2017), challenge this assumption somewhat, as we will later see in section 3.2.3). One first needs to suitably represent—or *vectorise*—persistence diagrams (PDs) in order to use them for downstream machine learning tasks. There are two predominant strategies for facilitating the integration of topological features into machine learning algorithms, namely (i) different representations that ideally give rise to feature vectors, and (ii) kernel-based methods that permit the integration into certain classifiers. Notice that these two strategies are not necessarily exclusionary; some representations, for example, also give rise to a kernel-based method.

Representations and kernel-based methods should ideally be efficiently computable, satisfy similar stability properties as the persistence diagrams themselves—hence exhibiting robustness properties with respect to noise—as well as provide some interpretable features. The stability of such representations is based on the fundamental stability theorem by Cohen-Steiner et al. (2007). In recent years, a multitude of suitable representation methods have been introduced; we present a selection thereof, focusing on representations that have already been used in machine learning contexts. As a somewhat broad categorisation, we observe that persistence diagrams are often mapped into an auxiliary *vector space*, e.g., by discretisation (Anirudh et al., 2016; Adams et al., 2017), or by mapping into a (Banach- or Hilbert-) *function space* (Chazal et al., 2014; Bubenik, 2015; Di Fabio and Ferri, 2015). Alternatively, there are several *kernel methods* (Reininghaus et al., 2015; Carrière et al., 2017; Kusano et al., 2018) that enable the efficient calculation of a similarity measure between persistence diagrams. Representations and kernel-based methods fall into the category of what we denote “observational” methods. The only exception is given by PersLay (Carrière et al., 2020), which informs the layers of the model and thus is an “interventional” method.

**Figure 5:**
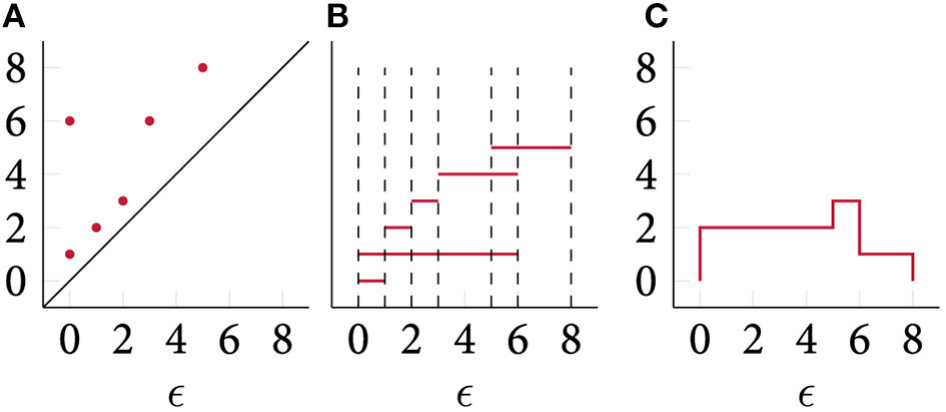
A persistence diagram **(A)**, its persistence barcode **(B)**, and its corresponding Betti curve **(C)**. Notice that the *interpretation* of the axes of different plots is different, hence we exclude labels for the barcode representation.

Arguably the most simple form of employing topological descriptors in machine learning tasks uses *summary statistics*, such as the total persistence of a persistence diagram (Cohen-Steiner et al., 2010), its *p*-norm (Chen and Edelsbrunner, 2011), or its persistent entropy (Atienza et al., 2019), i.e., the Shannon entropy of the individual persistence values in a diagram. While all of these approaches result in scalar-valued summary statistics, they are often not directly applicable to complex machine learning tasks, which require more expressive representations. We note, however, that such statistics give rise to hypothesis testing (Blumberg et al., 2014) based on topological information and we envision that this field will become more prominent as topological features find their use case for data analysis. A simple and stable representation of persistence diagrams, suitable for machine learning tasks, is provided by what are commonly called *Betti curves*. Given a persistence diagram D, and a weight function *w* : ℝ^2^ → ℝ, its Betti curve is the function β : ℝ → ℝ defined by

(1) $$\begin{array}{l} \beta \left(t\right)\;:\;=\underset{\left(b,d\right)\in D}{\sum }w\left(b,d\right)\cdot {𝟙}_{\left[b,d\right]}\left(t\right), \end{array}$$

where

(2) $$\begin{array}{l} {𝟙}_{\left[b,d\right]}\left(t\right)\;:\;=\{\begin{array}{ll} 1, & \text{if }t\in \left[b,d\right]\\ 0, & \text{else} \end{array} \end{array}$$

is the indicator function. The Betti curve was often informally used to analyse data (Umeda, 2017); recently, Rieck et al. (2020a) provided a summarising description of their features. Figure 5 depicts a simple illustration of the calculation of Betti curves. Betti curves are advantageous because they permit the calculation of a *mean* curve, next to providing an easy-to-evaluate distance and kernel method. Chevyrev et al. (2018) used this representation—and related “paths” derived from a persistence diagram and its representations—to solve classification tasks, using random forests and support vector machine classifiers. One drawback of the Betti curves is their limited expressive power. Being a summary statistic of a persistence diagram, the mapping from a diagram to a curve is not injective; moreover, the curve only contains *counts* of topological features and does not permit tracking single features, for example.

A more fundamental technique, developed by Carrière et al. (2015), *directly* generates a high-dimensional feature vector from a persistence diagram. The main idea is to obtain a vector representation of some persistence diagram D based on the distribution of pairwise distances of its elements, including points on the diagonal Δ: = {(*x, x*)|*x* ∈ ℝ} ⊂ ℝ^2^. More precisely, for each pair (*p, q*) of points in D, they compute *m*(*p, q*): = min{*d*_∞_(*p, q*), *d*_∞_(*p*, Δ), *d*_∞_(*q*, Δ)} and associate to D the vector of these values, sorted in descending order. As persistence diagrams may be of different sizes, they enlarge each of these vectors by zeros so that its length matches the length of the longest vector in the set. Hence, the set of persistence diagrams one considers needs to be fixed a priori. This vectorisation does not necessarily scale well to large data sets, but it can provide a good baseline to furnish *any* machine learning classifier—including a neural network—with simple topology-based feature vectors. The use of this technique appears to be restricted at present; we hope that our article will help increase its adoption.

**Figure 6:**
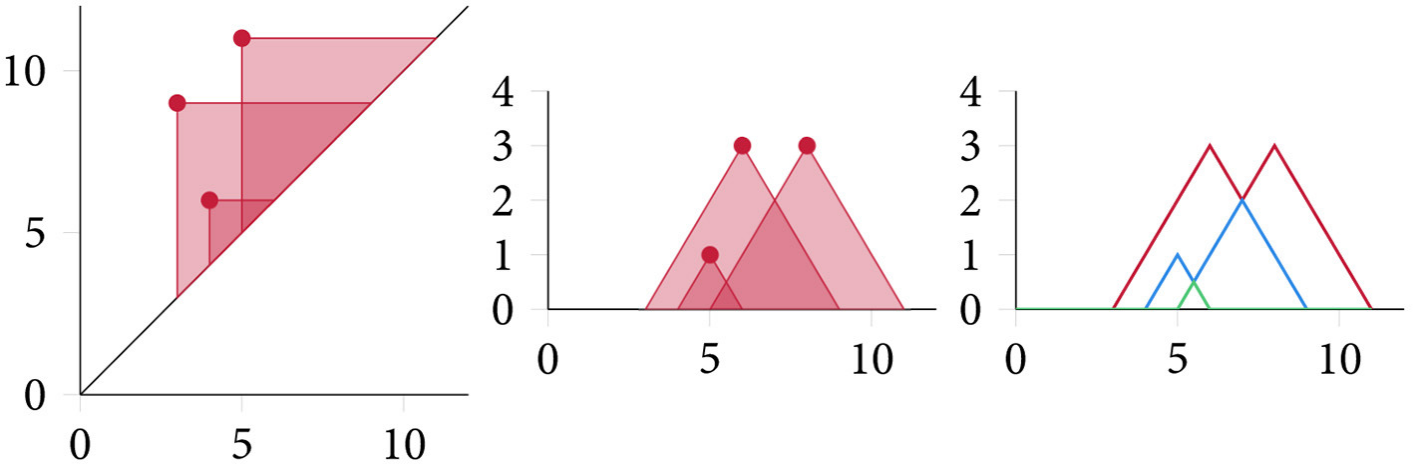
Computing a *persistence landscape* involves calculating the “area of influence” of each topological feature in a persistence diagram. Each connected shaded region with at least *k* intersections forms the basis of the *k*-th persistence landscape, which can be obtained by “peeling off” layers in an iterative fashion.

As a somewhat more complicated, but also more expressive, representation, Bubenik (2015) introduced topological descriptors called *persistence landscapes* that map persistence diagrams into a (Banach or Hilbert) function space in an invertible manner that satisfies stability properties with respect to the bottleneck distance of PDs. The *persistence landscape* λ : ℕ × ℝ → ℝ of a PD $D={\left\{\left(b_{i},d_{i}\right)\right\}}_{i\in I}$ can be defined in the following way. For *b* < *d*, we consider the auxiliary function *f*_(*b, d*)_(*t*): = max{0, min{*t* − *b, d* − *t*}} and define the persistence landscape as

$$\lambda \left(k,t\right)\;:\;=\text{kmax}{\left\{f_{\left(b_{i},d_{i}\right)}\left(t\right)\right\}}_{i\in I},$$

where kmax denotes the *k*-th largest element of the set. In addition to injectivity and stability, persistence landscapes do not require any choice of auxiliary parameters in their construction (see Figure 6 for a depiction of the persistence landscape computation process). They also afford various summary statistics, such as a norm calculation as well the calculation of both a kernel and a distance measure, making them a versatile representation of topological features. While, persistence landscapes have seen applications in time series analysis (Stolz et al., 2017), their most successful integration into machine learning algorithms is provided in the form of a new *layer*: persistence landscapes form the basis of a robust (with respect to noise) topological layer for deep neural networks, which is differentiable with respect to its inputs, the so-called PLLay (persistence landscape based topological layer) established in Kim et al. (2020). This layer exhibits good performance in image classification tasks as well as orbit classification, where it is shown to provide new state-of-the-art performance. We note that persistence landscapes are often considered in a vectorised form, which is obtained through binning their domain. While this is possible and useful for certain applications, we want to stress that the persistence landscape, as a lossless representation, should ideally be treated as such. The calculation of persistence landscapes imposes additional computational complexity, but the empirical performance reported by Kim et al. (2020) suggests that the landscapes are well-suited as a feature descriptor.

The *persistence images* (PIs), introduced by Adams et al. (2017), constitute an elegant hierarchical vectorisation step, representing a PD as a vector through the following steps. First the PD D is transformed from “birth–death”-coordinates into “birth–persistence”-coordinates via the transformation

$$T\;:\;{\mathbb{R}}^{2}\to {\mathbb{R}}^{2}\;:\;\left(x,y\right)\mapsto \left(x,y-x\right).$$

Next, for each *u* ∈ ℝ^2^ a differentiable probability density ϕ_*u*_ on ℝ^2^ is chosen (the standard choice being a normalised symmetric Gaussian with 𝔼[ϕ_*u*_] = *u*), as well as a weighting function $f:{\mathbb{R}}^{2}\to {\mathbb{R}}_{\geq 0}^{2}$ satisfying *f*|_{0} × ℝ_ ≡ 0. Additionally one chooses a discretisation of a relevant subdomain of ℝ^2^ by a standard grid. Each region *R* of this grid then corresponds to a pixel in the persistence image with value given by

$$\int_{R}\underset{u\in T\left(D\right)}{\sum }f\left(u\right)\phi_{u}\left(z\right)\text{d}z.$$

**Figure 7:**
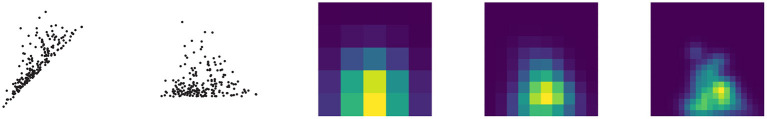
A persistence image arises as a discretisation of the density function (with appropriate weights) supported on a persistence diagram. It permits the calculation of an increasingly better-resolved sequence of images, which may be directly used as feature vectors.

In the process of generating persistence images, there are three non-canonical choices to be made. First, the choice of the weighting function, which is often chosen to emphasise features in the PD with large persistence value, next the distributions ϕ_*u*_, and lastly the resolution of the discretisation grid. Adams et al. (2017) prove that PIs are stable with respect to the 1-Wasserstein distance between persistence diagrams. Figure 7 illustrates their calculation. Persistence images are highly flexible and are often employed to make a classifier “topology-aware” to some extent (Zhao and Wang, 2019; Carrière and Blumberg, 2020; Rieck et al., 2020b). A paper by Zhao and Wang (2019), for instance, showcases their utility for graph classification. Interestingly, this paper constitutes also one of the few interventional approaches that employ extrinsic topological features; specifically, the authors use pre-defined filtrations to obtain graph-based persistence diagrams, and learn task-based weights for individual “pixels” (or “cells”) in the diagram. This approach is seen to surpass several graph classification algorithms on standard benchmark data sets—a remarkable feat, considering that the method does not employ any label information. The main drawbacks of persistence images are their quadratic storage and computation complexity, as well as the choice of appropriate parameters. While recent work found them to be remarkably stable in practice with respect to the Gaussian kernel parameters (Rieck et al., 2020b), there are no guidelines for picking such hyperparameters, necessitating a (cross-validated) grid search, for instance.

#### 3.2.2. Kernel-Based Representations

As an alternative to the previously-discussed representations, we now want to briefly focus on persistence diagrams again. The space of persistence diagrams can be endowed with metrics, such as the bottleneck distance. However, there is no natural Hilbert space structure on it, and such metrics tend to be computationally prohibitive or require the use of complex approximation algorithms (Kerber et al., 2017). Kernel methods provide a way of implicitly introducing such a Hilbert space structure to which persistence diagrams can be mapped via the feature map of the kernel. This then allows for a downstream use in machine learning models. To be more specific, given a set *X*, a function *k* : *X* × *X* → ℝ is called a (positive definite) *kernel* if there exists a Hilbert space $H_{k}$ together with a *feature map* $\phi :X\to H_{k}$ such that $k\left(x_{1},x_{2}\right)={\langle \phi \left(x_{1}\right),\phi \left(x_{2}\right)\rangle }_{H_{k}}$ for all *x*_1_, *x*_2_ ∈ *X*. Thus, by defining a kernel on the set of persistence diagrams, one obtains a vector representation via the feature map. However, in order for such a kernel to be useful in practice, it should additionally preserve the metric stability properties of persistence diagrams. Some pertinent examples of the kernel method are the following. Reininghaus et al. (2015) define a kernel on the set of persistence diagrams that is stable with respect to the 1-Wasserstein distance (Villani, 2009). The kernel is based on the idea of heat diffusion on a persistence diagram and offers a feature map that can be discretised (in fact, there are interesting similarities to persistence images). It was subsequently shown to satisfy *universality* (Kwitt et al., 2015), a desirable property for a kernel to have because it implies suitability for hypothesis testing. The *sliced Wasserstein kernel*, which is metric-preserving, was introduced by Carrière et al. (2017). It is based on the idea of the sliced Wasserstein distance (Kolouri et al., 2016), which ensures positive definiteness of the kernel through low-dimensional projections. Kusano et al. (2018) propose *persistence weighted Gaussian kernels* that incorporate a weighting and satisfy stability results with respect to the bottleneck distance and the 1-Wasserstein distance. The expressive power of kernels is in contrast to their computational complexity. Naïve implementations scale quadratically in the number of points, thus impeding the use of kernels for persistence diagrams with a large number of points. Some mitigation strategies exist (Greengard and Strain, 1991; Rahimi and Recht, 2008), but have not been adopted by implementations so far (moreover, their use is not always applicable, necessitating additional research). Nevertheless, such kernels are attractive because they are *not* limited with respect to the input data. Most of the papers exhibit good performance for shape classification or segmentation tasks, as well as in orbit classification.

While most of the aforementioned kernels are used to directly compare persistence diagrams, there are also examples of kernels *based on* topological information. An interesting example is provided by Rieck et al. (2019a), who introduce the Persistent Weisfeiler–Lehman (P-WL) kernel for graphs. It computes topological features during a Weisfeiler–Lehman (WL) procedure. The WL procedure refers to an iterative scheme in which vertex label information is aggregated over the neighbours of each vertex, resulting in a label multiset. A perfect hashing scheme is now applied to every multiset and the graph is relabelled with the ensuing hashes. This process can be repeated until a pre-defined limit has been reached or until the labels do not change any more. While originally intended as a test for graph isomorphism, it turns out that there are non-isomorphic graphs that cannot be distinguished by the WL procedure. However, it turns out to be an exceptionally useful way of assessing the dissimilarity between two graphs in polynomial time, leading to the WL kernel framework (Shervashidze and Borgwardt, 2009; Shervashidze et al., 2011), which enjoys great popularity for graph learning tasks (Borgwardt et al., 2020; Kriege et al., 2020). The P-WL extension of WL is characterised by its capability to extract topological information of the graph with respect to the current node labelling for each WL iteration. This kernel is particularly notable since it constitutes the first (to our knowledge) method that imbues data-based labels into the calculation of persistent homology.

#### 3.2.3. Integrating Topological Descriptors Into Neural Networks

One of the seminal methods that built a bridge between modern machine learning techniques and TDA is a work by Hofer et al. (2017). Using a differentiable projection function for persistence diagrams (with learnable parameters), the authors demonstrate that persistence diagrams of a data set can be easily integrated into *any* deep learning architecture. While the primary focus of the paper lies on developing such a projection function, the authors demonstrate the general feasibility of topological descriptors in both shape and graph classification tasks. A follow-up publication (Hofer C. D. et al., 2019) discusses more theoretical requirements for learning representations of topological descriptors.

This approach, as well as the development of the “DeepSets” architecture (Zaheer et al., 2017), which makes deep learning methods capable of learning *sets*, i.e., unordered sequences of varying cardinalities, spurred the development of *layers* that can be easily integrated into a deep learning workflow. An excellent example of such a layer is Carrière et al. (2020), which employs extended persistence (Cohen-Steiner et al., 2009) and heat kernel signatures to *learn* a vectorisation of persistence diagrams suited to the learning task at hand. PersLay is a neural network layer, defined by

$$\text{PersLay}\left(D\right)\;:\;=\text{op}\left({\left\{w\left(p\right)\cdot \phi \left(p\right)\right\}}_{p\in D}\right),$$

where D is a persistence diagram, op is and permutation invariant mapping, *w* : ℝ^2^ → ℝ is a weight function and ϕ : ℝ^2^ → ℝ^*d*^ is a vector representation function. Its generic definition allows PersLay to subsume and recover many existing representations by appropriate choices of op and ϕ (Carrière et al., 2020).

### 3.3. Intrinsic Topological Features in Machine Learning

This section reviews methods that either incorporate topological information directly into the design of a machine learning model itself, or leverage topology to study aspects of such a model. We refer to such features as *intrinsic topological features*. The primary examples are regularisation techniques as well as techniques for analysing neural network architectures.

#### 3.3.1. Regularisation Techniques

As a recent example, Moor et al. (2020) propose a topological autoencoder, which aims to preserve topological features of the input data in low-dimensional representations. This is achieved via a regularisation term that incentivises the persistence diagrams of both the latent and input space to be topologically similar. This method acts on the level of mini-batches, treating each of them as a point cloud. Persistence diagrams are obtained from the Vietoris–Rips complex of each space. By tracking the simplices that are relevant for the creation and destruction of topological features, and by consistently mapping simplices to a given edge in the Vietoris–Rips complex, each filtration can be interpreted as a selection of distances from the full distance matrix of the point cloud. The proposed regularisation term then compares the “selected” distances in the data space with the corresponding distances in the latent space (and vice versa). Finally, this regularisation is differentiable under the assumption that the persistence diagram is discrete (i.e., for each of its points, there is an infinitesimal neighbourhood containing no other points). The scheme can thus be directly integrated into the end-to-end training of an autoencoder, making it aware of the topology in the data space. This work can also be considered as an extension of previous work by Hofer C. et al. (2019), who introduced a differentiable loss term for one-class learning that controls the topology of the latent space; in effect, their loss term enforces a preferred “scale” for topological features in the latent space. It does not have to harmonise topological features *across* different spaces. It turns out that an autoencoder trained with this loss term on unlabelled data can be used on other data sets for one-class learning. This hints at the fact that enforcing a certain topological structure can be beneficial for learning tasks; we will later see that such empirical observations can also be furnished with a theoretical underpinning.

An approach by Chen et al. (2019) takes a different perspective. The authors develop a measure of the *topological complexity* (in terms of connected components) of the classification boundary of a given classifier. Said topological information is then used for regularisation in order to force the topological complexity of the decision boundary to be simpler, containing fewer features of low persistence. Thus, topological information serves as a penalty during classification such that training the classifier itself can be improved. In contrast to the aforementioned approach, differentiability is obtained through a “surrogate” piecewise linear approximation of the classifier. The method is seen to yield competitive results and the authors observe that the method performs well even in the presence of label noise. Analysing the decision boundary of a classifier also turns out to be advantageous for *model selection*, as we will later see in section 3.3.2.

Hofer et al. (2020a) analyse more fundamental principles of regularisation by means of topological features. Specifically, they study regularisation in a regime of small sample sizes with over-parametrised neural networks. By developing a new topological constraint for per-class probability measures, mass concentration effects in the vicinity of the learned representations of training instances are observed, leading to overall improvements of generalisation performance. The authors observe that controlling topological properties of learned representations presents numerous avenues for future research. These theoretical findings validate the empirical improvements observed in previous works of this domain.

As a more involved example of methods that make use of intrinsic features, Zhao et al. (2020) include topological features of graph neighbourhoods into a standard graph neural network (GNN) architecture. Their method combines a shortest-path filtration with persistence images, which are subsequently compressed to a single scalar value using a multilayer perceptron. The resulting scalar is then used to re-weight the message passing scheme used in training the GNN, thus obtaining topologically-based representations of graph neighbourhoods. In contrast to the previously-described loss terms, this method is not end-to-end differentiable, though, because the conversion from persistence diagrams to persistence images involves non-continuous parameters, i.e., the image dimensions. Zhao et al. (2020) primarily propose this method for node classification tasks, but we hypothesise that other graph tasks would profit from the integration of topological features.

Last, to provide a somewhat complementary perspective to preceding work, a paper by Hofer et al. (2020b) discusses how to employ graph neural networks (GNNs) to *learn* an appropriate filtration in an end-to-end fashion. The authors demonstrate that a GNN can be used to successfully initialise a scalar-valued filtration function, which can then subsequently be trained under mild assumptions (specifically, injectivity at the vertices of the graph needs to hold). The learned filtration turns out to surpass fixed filtrations combined with a persistent homology baseline, thus demonstrating the benefits of making topological representations differentiable—and thus *trainable*.

#### 3.3.2. Model Analysis

Shifting our view from regularisation techniques, topological analysis has been applied to evaluate generative adversarial networks (GANs). A GAN (Goodfellow et al., 2014) is comprised of two sub-networks, a generator and a discriminator. Given a data distribution *P*_data_, the generators objective is to learn a distribution *P*_model_ with the same statistics, whereas the discriminator learns to distinguish generated samples from actual data samples. The topological evaluation of GANs is motivated by the manifold hypothesis (Fefferman et al., 2013), which poses that a data distribution *P*_data_ is sampled from an underlying manifold $M_{\text{data}}$. The idea is to assess the topological similarity of $M_{\text{data}}$ and the underlying manifold $M_{\text{model}}$ of the model generated distribution *P*_model_. Based on the persistent homology of witness complexes, Khrulkov and Oseledets (2018) introduce the *Geometry Score*, which is a similarity measure of the topologies of $M_{\text{data}}$ and $M_{\text{model}}$ and can be used to evaluate generative models. Later work by Zhou et al. (2021) generalises this approach and additionally extends it to the disentanglement evaluation of generative models in unsupervised settings.

In a different direction, the topological analysis of the intrinsic structure of a classifier, such as a neural network, makes it possible to improve a variety of tasks. This includes the analysis of training behaviour as well as model selection—or *architecture selection* in the case of neural networks.

While the literature dedicated to the better understanding of deep neural networks has typically focused on its functional properties, Rieck et al. (2019b) took a different perspective to focus on the graph structure of a neural network. Specifically, they treat a (feed-forward) neural network as a stack of bipartite graphs. From this view, they propose “neural persistence,” a complexity measure which summarizes topological features that arise when calculating a filtration of the neural network graph where the filtration weights are given by the network parameters. They showed that neural persistence can distinguish between well-trained and badly-trained (i.e., diverged) networks. This measure is oblivious to the functional behaviour of the underlying network, but only focuses on its (weighted) *structure*. Nevertheless, Rieck et al. (2019b) showed that it can be used for guiding early stopping solely based on topological properties of the neural network, potentially saving validation data used for the early stopping decision.

Ramamurthy et al. (2019) employ labelled variants of simplicial complexes, such as a labelled Vietoris–Rips complex, to analyse the decision boundary (i.e., classification boundary) of a given classifier. The authors are able to provide theoretical guarantees that the correct homology of a decision boundary can be recovered from samples, thus paving the way for an efficient approximation scheme that incorporates local scale estimates of the data set. Such a construction is required because the density of available samples is not guaranteed to be uniform, leading to simplicial complexes with spurious simplices in high-density regions, while running the risk of “undersampling” low-density regions. Next to “matching” models based on the *Decision Boundary Topological Complexity* (DBTC) score, Ramamurthy et al. (2019) also enable matching data sets to pre-trained models. The underlying assumption is that a model that closely mimics the topological complexity of a data set is presumably a better candidate for this particular data set.

Gabrielsson and Carlsson (2019) utilise topological data analysis to analyse topological information encoded in the weights of convolutional neural networks (CNNs). They show that the weights of convolutional layers encode simple global structures which dynamically change during training of the network and correlate with the network's ability to generalise to unseen data. Moreover, they find that topological information on the trained weights of a network can lead to improvements in training efficiency and reflect the generality of the data set on which the training was performed.

## 4. Outlook and Challenges

This survey provided a glimpse of the nascent field of *topological machine learning*. We categorised existing work depending on its intention (interventional vs. observational) and according to what type of topological features are being calculated (extrinsic vs. intrinsic), finding that most extrinsic approaches are observational, i.e., they do not inform the choice of model afterwards, while most intrinsic approaches are interventional, i.e., they result in changes to the choice of model or its architecture.

Numerous avenues for future research exist. Of the utmost importance is the improvement of the “software ecosystem.” Software libraries such as GUDHI (Maria et al., 2014) and giotto-tda (Tauzin et al., 2021) are vital ingredients for increasing the adoption of TDA methods, but we envision that there is a specific niche for libraries that integrate *directly* with machine learning frameworks such as pytorch. This will make it easier to disseminate knowledge and inspire more research. A challenge that the community yet has to overcome involves the overall scalability of methods, though. While certain improvements on the level of filtrations are being made (Sheehy, 2013; Cavanna et al., 2015), those improvements have yet to be integrated into existing algorithms. A more fundamental question is to what extent TDA has to rely on “isotropic” complexes such as the Vietoris–Rips complex, and whether scale-dependent complexes that incorporate sparsity can be developed.

On the side of applications, we note that several papers already target problems such as graph classification, but they are primarily based on fixed filtrations (with the notable exception of Hofer et al. (2020b), who learn a filtration end-to-end). We envision that future work could target more involved scenarios, such as the creation of “hybrid” GNNs, and the use of end-to-end differentiable features for other graph tasks, such as node classification, link prediction, or community detection.

As another upcoming topic, we think that the analysis of time-varying data sets using topology-based methods is long overdue. With initial work by Cohen-Steiner et al. (2006) on time-varying topological descriptors providing a theoretical foundation, there are nevertheless few topology-based approaches that address time series classification or time series analysis. Several—theoretical and practical—aspects for such an endeavour are addressed by Perea et al. (2015), who develop a persistence-based method for quantifying periodicity in time series. The method is based on the fundamental embedding theorem by Takens (1981) and is combined with a sliding window approach. Future work could build on such approaches, or find other ways to characterise time-series, for instance based on complex networks (Lacasa et al., 2008). This could pave the road toward novel applications of TDA such as anomaly detection.

## Author Contributions

FH, MM, and BR performed the literature search and revised the draft. FH and BR drafted the original manuscript. All authors contributed to the article and approved the submitted version.

## Conflict of Interest

The authors declare that the research was conducted in the absence of any commercial or financial relationships that could be construed as a potential conflict of interest.
